# Persistent sleep disturbances after mild traumatic brain injury: A prospective multimodal assessment with actigraphy and hormonal biomarkers

**DOI:** 10.1016/j.bas.2026.106014

**Published:** 2026-03-23

**Authors:** Igor Paredes, Ana Maria Castaño-Leon, Victoria Cunha Alves, Cristina Sánchez Carabias, Juan Delgado-Fernandez, Luis Miguel Moreno, Guillermo García-Posadas, Mónica Maldonado Luna, Andreea Emanuela Baciu, Leandro Tosi, Alfonso Lagares

**Affiliations:** a Instituto de Investigación Sanitaria Hospital 12 de Octubre (imas12), Madrid, Spain; b Department of Surgery, Faculty of Medicine, Universidad Complutense de Madrid, Madrid, Spain; c Neurosurgery Department, Hospital Universitario 12 de Octubre, Madrid, Spain

**Keywords:** Mild traumatic brain injury, Sleep disturbance, Actigraphy, Melatonin, Orexin, Predictive factors

## Abstract

**Introduction:**

Sleep disturbance is among the most frequent and disabling persistent post-concussive symptoms (PPCS) following mild traumatic brain injury (mTBI). However, the relationship between subjective complaints, actigraphic parameters, and neurohormonal markers remains poorly characterized.

**Research question:**

To determine the frequency, severity, and physiological correlates of sleep disturbance after mTBI, and to identify early objective predictors of persistent symptoms at three months.

**Material and methods:**

A prospective study including 51 patients with mTBI and 39 age- and sex-matched controls. Sleep was assessed at 1 week and 3 months using the Pittsburgh Sleep Quality Index (PSQI), Epworth Sleepiness Scale (ESS), 7-day wrist actigraphy (a wearable motion sensor to continuously monitor sleep-wake cycles), and plasma/salivary melatonin and orexin A/B levels. Group comparisons and within-subject changes were analyzed using parametric or non-parametric tests; correlations were assessed using Spearman's ρ.

**Results:**

At 1 week, 73.9% of patients met criteria for subjective sleep disturbance, with 48.6% remaining symptomatic at 3 months. Compared with controls, mTBI patients showed reduced sleep efficiency and increased wake after sleep onset. Higher ESS and PSQI scores correlated with longer sleep latency and more awakenings (ρ = 0.37–0.47; p < 0.05). Evening orexin A levels were elevated in symptomatic patients (p = 0.048). Shorter minimal sleep latency was the only independent predictor of persistent disturbance.

**Discussion and conclusion:**

Sleep disturbances after mTBI are common and partly reflected in objective alterations of sleep continuity. Early actigraphic evidence of hyperarousal may predict persistent symptoms. These findings support multimodal assessment to identify individuals at risk for chronic post-traumatic sleep disturbance.

## Highlights

• Persistent sleep disturbances are common after mild traumatic brain injury.
• Actigraphy shows reduced sleep efficiency and continuity in mTBI patients.
• Early actigraphic hyperarousal predicts persistent sleep symptoms at 3 months.
• Elevated acute evening orexin A levels associate with sleep disturbances.

## Abbreviations

CI: confidence interval
ESS: Epworth Sleepiness Scale
IQR: interquartile range
IRB: Institutional Review Board
LH: lateral hypothalamus
LOC: loss of consciousness
mTBI: mild traumatic brain injury
PPCS: persistent post-concussive symptoms
PSQI: Pittsburgh Sleep Quality Index
PTA: post-traumatic amnesia
ROI: region of interest
SCN: suprachiasmatic nucleus
SD: standard deviation
TIB: time in bed
TST: total sleep time
VLPO: ventrolateral preoptic nucleus
WASO: wake after sleep onset
ρ: Spearman's rho

## 1 Introduction

Mild traumatic brain injury (mTBI) is among the most common neurological conditions worldwide, accounting for the majority of traumatic brain injuries seen in emergency departments. While most patients recover within weeks, a substantial proportion experience persistent symptoms—collectively termed persistent post-concussive symptoms (PPCS)—that can significantly impact quality of life and occupational performance. Sleep disturbances, including insomnia, hypersomnia, excessive daytime sleepiness, and circadian rhythm misalignment, are among the most prevalent and disabling PPCS after mTBI, second only to post-traumatic headache (Mathias and Alvaro, 2012).

The underlying mechanisms of post-traumatic sleep dysfunction remain poorly understood, but proposed pathways include disruption of circadian regulation and injury to sleep–wake regulatory centers such as the hypothalamus (Carskadon et al., 1976; Grima et al., 2016; Willie et al., 2001). Specifically, the interplay between sleep-promoting centers, such as the ventrolateral preoptic nucleus, and wake-stabilizing neuropeptides like orexin (hypocretin), may be compromised following shearing forces inherent to mTBI(5, 6). Similarly, diffuse axonal injury or metabolic dysfunction may impair the suprachiasmatic nucleus—the master circadian pacemaker—leading to altered melatonin profiles and circadian misalignment (Grima et al., 2016; Comai and Gobbi, 2024) Despite this theoretical framework, combined clinical assessments investigating these hormonal biomarkers alongside sleep continuity remain scarce.

Furthermore, the agreement between subjective complaints and objective measures of sleep in mTBI appears to be limited. Several reports describe a poor correlation between self-reported symptoms (e.g., insomnia, daytime sleepiness) and objective data derived from actigraphy or polysomnography (Buysse et al., 1989; Harvey and Tang, 2012; Raikes et al., 2019). This subjective-objective mismatch complicates clinical management, raising questions about whether self-reported poor sleep reflects true physiological fragmentation, altered sleep perception, or the influence of comorbid psychological distress, such as pain or mood alterations (Harvey and Tang, 2012; Chaput et al., 2009). Consequently, there is a critical need to utilize a multimodal approach to fully characterize post-traumatic sleep pathology.

Therefore, the present study was designed to test the following exploratory hypotheses (Mathias and Alvaro, 2012): patients with mTBI will exhibit a higher frequency and severity of sleep disturbances compared to matched controls, both in the acute phase and at 3 months post-injury (Carskadon et al., 1976); there will be a correlation between subjective sleep complaints and objective actigraphic parameters; and (Grima et al., 2016) specific early objective sleep metrics and/or hormonal profiles (melatonin and orexin) can serve as predictors for the persistence of sleep disturbances at 3 months.

## 2 Materials and methods

### 2.1 Study design and setting

We conducted a prospective observational cohort study at Hospital Universitario 12 de Octubre, a level I trauma center in Madrid, Spain, serving a catchment area of approximately 600,000 inhabitants. Recruitment took place between September 2021 and September 2024. The study protocol was approved by the local Ethics Committee (N° CEIm: 21/391), and all participants provided written informed consent.

### 2.2 Participants

Two cohorts were enrolled:

• **mTBI group** – Patients ≥16 years old presenting within 24 h of injury with a Glasgow Coma Scale (GCS) score of 13–15, meeting institutional criteria for cranial CT acquisition, aligned with the Canadian CT Head Rules (Stiell et al., 2001). Eligible patients must have had either an abnormal CT scan or clinical/anamnestic features requiring ≥24 h of observation (e.g., post-traumatic amnesia >30 min, loss of consciousness >5 min or persistent disorientation).
• **Control group** – Age- and sex-matched volunteers without a history of TBI or diagnosed sleep disorders. Controls were recruited from hospital staff (strictly excluding individuals performing night shifts during the study period) and relatives of the patients.

**Exclusion criteria** for both groups included: prior diagnosis of primary sleep disorders, obesity (BMI >30 kg/m^2^, to minimize the confounding effect of undiagnosed obstructive sleep apnea), recent transmeridian travel or shift work (within 6 weeks), current use of hypnotic medications, unstable medical or psychiatric illness, previous mTBI fulfilling the inclusion criteria in the previous 3 months, and inability to complete assessments due to sensory or language limitations.

### 2.3 Subjective sleep assessment

At 1 week and 3 months post-injury, participants completed the Pittsburgh Sleep Quality Index (PSQI) and Epworth Sleepiness Scale (ESS). Operational definition and cut-offs: In line with original descriptions and extensive validation, ESS >10 was considered indicative of excessive daytime sleepiness, and PSQI >5 indicative of poor sleep quality. These thresholds have shown acceptable sensitivity, specificity, and stability across clinical and population-based samples (Buysse et al., 1989, 2008; Kendzerska et al., 2014; Mollayeva et al., 2016; Thorarinsdottir et al., 2019; Hinz et al., 2017; Johns, 1991; Knutson et al., 2006). As the ESS (daytime sleep propensity) and PSQI (nocturnal sleep quality) capture complementary constructs, their combined use improves detection of distinct sleep disturbance phenotypes; we therefore defined “subjective sleep disturbance” as meeting either cut-off (Chaput et al., 2009; Buysse et al., 2008; Kendzerska et al., 2014; Mollayeva et al., 2016; Thorarinsdottir et al., 2019; Baumann et al., 2007).

### 2.4 Objective sleep assessment (actigraphy)

Participants wore an Actiwatch 2 on the non-dominant wrist for 7 days (5-s epochs), analyzed with Actiware v6.3 using the validated sleep–wake algorithm. Bedtime and wake-time were marked with the event button; when missing/implausible, lights-off/on and activity profiles were used to impute intervals per software guidelines.

The following variables were extracted:

• Time in bed (min)
• Total sleep time (min)
• Sleep latency (min)
• Sleep efficiency (%) — defined as the ratio of total sleep time to time in bed × 100
• Wake after sleep onset (WASO, min)
• Number of awakenings (events). (Note that due to our highly sensitive 5-s epoch resolution, an event is triggered by brief micro-movements across consecutive epochs, resulting in higher absolute event numbers per night compared to standard 30-s epoch scoring).

### 2.5 Biological samples and hormonal assays

Blood and saliva samples were collected within the first 24 h after injury (morning: 07:00–09:00; evening: 19:00–21:00) and again in the morning at 1 week and 3 months. Blood samples were allowed to clot and centrifuged at 1000 g for 30 min. Unstimulated saliva samples were collected in sodium azide-precoated Salivette® (Cat No. 51.1534, Sarstedt, Germany), centrifuged at 1000 g for 2 min, and treated with a complete protease inhibitor cocktail (Cat No. 11836153001, Roche, Switzerland). Samples were aliquoted and stored at −80 °C until analysis. The quantitative determination of total protein concentration in saliva samples was performed using the Pierce BCA Protein Assay Kit (Cat No. 23227, Thermo Scientific, USA), and samples were normalized to equal concentration (1 mg/mL) prior to biomarker quantitation. Serum and salivary melatonin and serum orexin-A and orexin-B concentrations were quantified using commercially available ELISA kits, following the manufacturer's protocols (Cat Nos. ab283259 and ab283258, abcam, Netherlands; Cat Nos. CSB-E08859h and CSB-E09863h, Cusabio, USA). Serum and salivary melatonin were successfully quantified. Orexin A and B were assayed in serum; but could not be reliably analyzed in saliva due to assay sensitivity limitations.

### 2.6 Outcome definitions

• **Subjective sleep disturbance**: PSQI score >5 or ESS score >10 at the 1-week assessment.
• **Persistent sleep disturbance**: same criteria met at the 3-month follow-up.
• **Objective sleep disturbance**: actigraphy showing sleep efficiency <85% or sleep latency >30 min.

### 2.7 Statistical analysis

An a priori sample size calculation was performed based on expected differences in morning melatonin concentrations. Assuming an estimated variance of 77.42 based on previous literature, and aiming to detect a minimal difference of 3.5 pg/mL between patients with and without sleep disturbances (with 90% statistical power, a 95% confidence level, and allowing for a 10% dropout rate), the target sample size was set at 132 patients. However, due to stringent inclusion criteria, the high logistical burden of the multimodal protocol (which required 7-day actigraphy, multiple hospital return visits, and biological sampling), and the impact of the COVID-19 pandemic on clinical research participation, the final recruited sample was limited to 51 patients. Consequently, analyses were conducted on the available sample, recognizing the study as exploratory and potentially underpowered for detecting subtle biomarker differences.

Analyses were performed using SPSS (IBM Corp., v29) and RStudio (Posit PBC). Continuous variables (e.g., actigraphy parameters, hormone concentrations, PSQI, and ESS scores) were first assessed for normality using the Kolmogorov–Smirnov test with Lilliefors correction. To compare differences between independent groups (e.g., mTBI vs. controls, or persistent vs. non-persistent disturbances), Student's t-tests were utilized for normally distributed variables, while the Mann–Whitney *U* test was applied to non-normally distributed data (such as specific hormone levels). Categorical variables (e.g., sex, presence of CT abnormalities) were compared using χ^2^ or Fisher's exact test. To address the magnitude of the findings, effect sizes were calculated for all group comparisons (Cohen's d for parametric tests, r for non-parametric tests, and Cramer's V for categorical comparisons).

To evaluate the relationship between subjective questionnaires and objective actigraphy metrics, Pearson's or Spearman's correlation coefficients (ρ) were calculated depending on data distribution. Finally, for the exploratory prediction of persistent sleep disturbance at 3 months, a forward stepwise logistic regression guided by Akaike's Information Criterion (AIC) was conducted. The model included baseline and 1-week candidate variables (objective actigraphy, subjective questionnaires, hormonal assays, and basic laboratory parameters) that demonstrated a potential association (p < 0.10) in univariate analyses. The logistic regression model was performed using a complete-case approach. Patients with missing values in any of the candidate predictor variables or in the 3-month outcome were excluded from the multivariable analysis, resulting in a final sample of 40 patients (Missing data were due mainly to incomplete actigraphy recordings or unavailable hormonal measurements). Statistical significance was set at α = 0.05.

## 3 Results

A total of 90 participants were included, comprising 51 patients with mild traumatic brain injury (mTBI) and 39 healthy controls. The mean age was 45 ± 22 years in the mTBI group and 40 ± 16 years in controls, with no statistically significant difference (p = 0.12). Women accounted for 31.4% of the mTBI group and 43.6% of controls (p = 0.21).

In the mTBI cohort, 12 patients (23.5%) had pathological CT findings. All presented traumatic subarachnoid hemorrhage, and one case (8.3%) also had an acute subdural hematoma. The remaining 39 patients met the study's inclusion criteria based on clinical or anamnestic features in the absence of radiological abnormalities. Regarding injury severity markers, loss of consciousness (LOC) was absent in 26 patients (51.0%), lasted 1–29 min in 9 (17.6%), 30–59 min in 2 (3.9%), and was unknown in 14 (27.5%). Post-traumatic amnesia (PTA) was absent in 31 patients (60.8%), lasted <1 min in 2 (3.9%), 1–29 min in 8 (15.7%), 30–59 min in 5 (9.8%), and was unknown in 5 (9.8%).

### 3.1 Subjective sleep disturbances

At 1 week post-injury, 73.9% of mTBI patients met criteria for subjective sleep disturbance (PSQI >5 or ESS >10), with mean ESS 9.3 ± 4.7 (n = 47) and PSQI 6.7 ± 3.4 (n = 45). At 3 months, proportion decreased to 53.8%, with mean ESS 8.7 ± 5.9 (n = 37) and PSQI 5.1 ± 2.9 (n = 39). A persistent disturbance was observed in 18 patients, while 2 developed a de novo disturbance at 3 months.

In the control group, 68.2% presented pathological ESS or PSQI scores at baseline, with a mean ESS of 7.6 ± 3.9 (n = 24) and PSQI of 7.0 ± 3.7 (n = 22). When compared with controls, mTBI patients did not exhibit statistically significant differences in mean ESS scores at 1 week (9.3 vs. 7.6, p = 0.07) or at 3 months (8.7 vs. 7.6, p = 0.21). Similarly, no significant differences were observed in PSQI scores at 1 week (6.7 vs. 7.0, p = 0.71). Therefore, subjective baseline sleep complaints were comparable between the mTBI cohort and the selected matched controls.

### 3.2 Objective sleep (actigraphy)

At 1 week post-injury, actigraphic assessments revealed significant differences in sleep continuity between mTBI patients and matched controls. Patients spent significantly more time in bed and had a longer total sleep time. However, sleep quality was objectively worse in the mTBI cohort, evidenced by significantly lower sleep efficiency, increased time awake after sleep onset (WASO), and a higher number of awakening events compared to controls. Sleep latency was longer in patients, though this difference did not reach statistical significance.

**Fig. 1:**
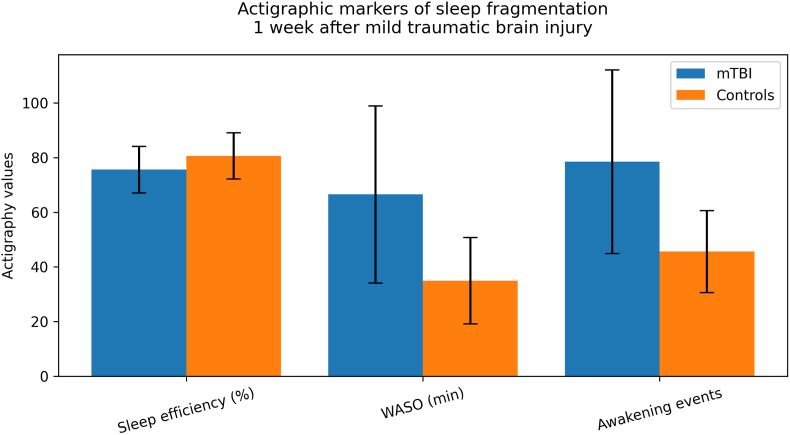
**Actigraphic markers of sleep fragmentation at 1 week after mild traumatic brain injury.** Sleep efficiency was significantly lower in patients with mild traumatic brain injury (mTBI) compared with matched controls. Wake after sleep onset (WASO) was significantly higher in the mTBI cohort, indicating greater nocturnal sleep fragmentation. (The number of awakening events detected by actigraphy was also significantly higher in patients. Bars represent mean ± standard deviation from 7-day wrist actigraphy recordings.

At the 3-month follow-up, differences in time in bed, total sleep time, sleep latency, and overall sleep efficiency were no longer statistically significant between the two groups. Nevertheless, objective markers of sleep fragmentation persisted, as mTBI patients continued to exhibit significantly higher WASO and a greater number of awakening events than controls (Table 1, Fig. 1).

**Table 1:** Sleep Variables mTBI vs Controls.

Variable	mTBI (mean ± SD)	Controls (mean ± SD)	p-value	Effect Size (Cohen's d)
**Time in bed (min) - 1 week**	635.6 ± 185.1	508.0 ± 70.7	<0.001	0.87
**Total sleep time (min) - 1 week**	476.2 ± 124.9	409.2 ± 58.1	0.003	0.66
**Sleep latency (min) - 1 week**	51.5 ± 33.3	42.2 ± 33.2	0.213	0.28
**Sleep efficiency (%) - 1 week**	75.6 ± 8.5	80.6 ± 8.5	0.011	0.59
**Wake after sleep onset (WASO, min) - 1 week**	66.5 ± 32.4	35.0 ± 15.8	<0.001	1.19
**Awakening events - 1 week**	78.5 ± 33.6	45.6 ± 15.0	<0.001	1.21
**Time in bed (min) - 3 months**	544.3 ± 101.2	508.0 ± 70.7	0.079	0.41
**Total sleep time (min) - 3 months**	429.9 ± 61.4	409.2 ± 58.1	0.149	0.35
**Sleep latency (min) - 3 months**	42.2 ± 39.4	42.2 ± 33.2	0.998	0.00
**Sleep efficiency (%) - 3 months**	78.2 ± 11.5	80.6 ± 8.5	0.333	0.23
**Wake after sleep onset (WASO, min) - 3 months**	45.1 ± 23.9	35.0 ± 15.8	0.036	0.49
**Awakening events - 3 months**	57.3 ± 25.6	45.6 ± 15.0	0.019	0.54

Table 1. Actigraphy-derived sleep parameters in patients with mild traumatic brain injury (mTBI) and matched controls at 1 week and 3 months.Data are presented as mean ± standard deviation. Effect sizes are expressed as Cohen's d. WASO = wake after sleep onset.

### 3.3 Correlations between questionnaires and actigraphic parameters

At 1 week post-injury, patients with mild TBI exhibited significant correlations between subjective scales and actigraphy. Higher Epworth scores were significantly associated with longer sleep latency (ρ = 0.41, p = 0.03) and with a greater number of nocturnal awakenings (events categorized as wake; ρ = 0.46, p = 0.02). Similarly, higher Pittsburgh Sleep Quality Index (PSQI) total scores correlated negatively with actigraphy-derived sleep efficiency (ρ = −0.47, p = 0.01) and positively with the number of awakenings (events; ρ = 0.43, p = 0.02). These findings suggest that in patients with mild TBI, subjective complaints of poor sleep and daytime sleepiness are aligned with objective disruptions in sleep continuity and efficiency (Table 2).

**Table 2:** – Significant correlations between clinical scales and actigraphy in patients with mild TBI (Spearman's ρ).

Time point	Scale (subjective)	Actigraphy variable (objective)	Direction	ρ (Spearman)	p-value
1 week	Epworth Sleepiness Scale	Sleep latency (maximum)	Positive	0.41	0.03
1 week	Epworth Sleepiness Scale	Number of awakenings (events)	Positive	0.46	0.02
1 week	PSQI – Total score	Sleep efficiency (mean)	Negative	−0.47	0.01
1 week	PSQI – Total score	Number of awakenings (events)	Positive	0.43	0.02
3 months	Epworth Sleepiness Scale	Awakenings (contiguous)	Positive	0.42	0.010
3 months	Epworth Sleepiness Scale	Awakenings (events)	Positive	0.37	0.027
3 months	PSQI – Sleep duration	Time in bed	Negative	−0.41	0.011
3 months	PSQI – Sleep duration	Total sleep time	Negative	−0.40	0.015
3 months	PSQI – Sleep duration	Awakenings (events)	Negative	−0.42	0.009

Table 2. Significant correlations between subjective scales and actigraphy parameters at 1 week and 3 months post-injury (Spearman's ρ). Contiguous awakenings correspond to consecutive wake epochs detected by the actigraphy algorithm. Only significant correlations (p < 0.05) are shown. Positive correlations indicate that higher questionnaire scores are associated with longer or more fragmented sleep; negative correlations indicate that higher scores are associated with reduced sleep efficiency or duration.

**Table 3:** Variables associated with persistent subjective sleep disturbance.

Variable	Persistent disturbance (mean ± SD)	No persistent disturbance (mean ± SD)	p-value	Effect Size (Cohen's d)
**Minimal sleep latency (min) - 1 week**	3.08 ± 5.12	12.55 ± 13.16	0.004	0.91
**Mean sleep efficiency (%) - 1 week**	78.54 ± 6.34	75.04 ± 9.08	0.161	0.44
**Serum orexin A (morning) (pg/mL)**	0.294 ± 0.241	0.149 ± 0.090	0.041	0.83
**Serum orexin B (morning) (pg/mL)**	241.51 ± 60.88	175.30 ± 81.51	0.011	0.91
**Serum orexin A (evening) (pg/mL)**	0.315 ± 0.261	0.140 ± 0.094	0.020	0.93
**Serum Melatonin evening (pg/mL)**	69.87 ± 14.26	88.82 ± 40.86	0.071	0.60
**Glucose (mg/dL)**	103.88 ± 17.79	127.32 ± 24.60	0.003	1.07

Table 3. Baseline and 1-week variables associated with persistent subjective sleep disturbance at 3 months. Values are presented as mean ± standard deviation. Effect sizes are expressed as Cohen's d.

In the control group, no consistent associations between subjective questionnaires and actigraphy were detected.

At three months, mTBI cases also showed Epworth scores that correlated positively with objective measures of sleep fragmentation. Higher Epworth scores were associated with more nocturnal awakenings, both in time awake (ρ = 0.42, p = 0.010) and in wake events (ρ = 0.37, p = 0.027). Within the PSQI, the sleep duration component showed negative correlations with actigraphy-derived measures, including time in bed (ρ = −0.41, p = 0.011), total sleep time (ρ = −0.40, p = 0.015), and the number of awakenings (events; ρ = −0.42, p = 0.009). No significant correlations were found for the PSQI total score.

### 3.4 Hormonal assays (morning samples)

Analyses did not reveal significant differences between groups at 1 week or 3 months for serum or salivary melatonin concentrations. Regarding orexin levels, serum orexin A and B concentrations were comparable between patients and controls at 1 week. At 3 months, orexin A levels remained similar between groups; however, serum orexin B concentrations were significantly lower in mTBI patients compared with controls (218.46 ± 93.16 vs 272.65 ± 105.48; p = 0.041; Supplementary Table 1).

### 3.5 Within-patient/controls differences

At 1 week post-injury, mTBI patients classified with subjective sleep disturbance (pathological PSQI and/or ESS scores) exhibited significantly higher evening serum orexin A levels compared with non-disturbed patients (0.2299 ± 0.2194 vs. 0.1524 ± 0.0713 pg/mL; p = 0.048, unequal variances). Actigraphy-derived parameters, such as maximum sleep latency and sleep efficiency, did not differ significantly between these subgroups. In the control group, participants with subjective sleep disturbance showed no significant differences in actigraphy-derived total sleep time, sleep latency, or sleep efficiency compared to good sleepers. Although disturbed controls exhibited numerical differences in endocrine profiles (higher morning and salivary melatonin, higher serum orexin B, and lower serum orexin A), none of these differences reached statistical significance after correction.

### 3.6 Persistent sleep disturbance (3 months. Table 3)

When comparing patients with and without persistent subjective sleep disturbance at 3 months, those in the persistent group demonstrated a significantly shorter minimal sleep latency at 1 week (3.08 ± 5.12 vs. 12.55 ± 13.16 min; p = 0.004). Mean sleep efficiency at 1 week appeared slightly higher in the persistent group (78.54 ± 6.34% vs. 75.04 ± 9.08%), though this difference did not reach statistical significance (p = 0.161) and likely reflects sample variability rather than a true protective effect. Other actigraphic parameters at 1 week, including time in bed, total sleep time, time awake, and number of awakenings, did not differ significantly between the two groups (all p > 0.10). Similarly, no significant differences in actigraphic variables emerged at the 3-month follow-up (all p > 0.10). Furthermore, there were no significant differences between patients with and without persistent symptoms regarding age, sex, injury mechanism, or the presence of CT abnormalities.

### 3.7 Hormones (acute/first 24 h)

Regarding acute hormonal assays collected within the first 24 h, the persistent group exhibited significantly higher morning serum orexin A (p = 0.041), higher morning serum orexin B (p = 0.011), and higher evening serum orexin A levels (p = 0.020). Conversely, evening melatonin levels were lower in the persistent group, though not reaching statistical significance (69.87 ± 14.26 vs. 88.82 ± 40.86 pg/mL; p = 0.071), with morning melatonin displaying a similar non-significant pattern (79.12 ± 19.03 vs. 94.79 ± 31.60 pg/mL; p = 0.090).

### 3.8 Standard laboratory tests (acute)

Among standard acute laboratory tests, blood glucose levels were significantly lower in patients who later developed persistent disturbances (103.88 ± 17.79 vs. 127.32 ± 24.60 mg/dL; p = 0.003). No other analyzed parameters showed significant differences (all p > 0.10).

### 3.9 Exploratory prediction of persistent disturbance

A forward, AIC-guided logistic regression was conducted in the mTBI cohort (n = 40) including all baseline/1-week variables that showed p < 0.10 in univariate analyses: minimal sleep latency (1 week), mean sleep efficiency (1 week), serum orexin A (morning), serum orexin B (morning), serum orexin A (evening), serum melatonin (evening) and glucose. The final model retained minimal sleep latency at 1 week as the only independent predictor of persistent sleep disturbance at 3 months (OR per SD decrease = 0.25; 95 % CI 0.06–0.95; p = 0.041), with an AUC of 0.73. None of the hormonal or laboratory parameters improved model fit once minimal latency was included (all p > 0.10; ΔAIC <2).

## 4 Discussion

In this prospective cohort study, we combined subjective questionnaires (Pittsburgh Sleep Quality Index, Epworth Sleepiness Scale) (Buysse et al., 1989; Johns, 1991), actigraphy, and hormonal assays to investigate post-traumatic sleep disturbances following mTBI. At 3 months, more than half of the patients still met criteria for subjective sleep disturbance, with a persistent form observed in over one-third of the cohort. This aligns with prior evidence suggesting that sleep complaints after mTBI can remain clinically significant well beyond the acute phase, impacting recovery, cognition, and quality of life (Mathias and Alvaro, 2012; Baumann et al., 2007; Viola-Saltzman and Musleh, 2016). It is important to acknowledge that, based on our strict inclusion criteria (requiring abnormal CT findings or clinical features warranting >24 h of observation), our cohort likely represents the more severe end of the mild TBI spectrum, often referred to as “complicated mTBI.” This higher baseline severity may partially explain the elevated frequency of acute and persistent sleep disturbances observed in our sample compared to milder cohorts.

Actigraphy confirmed objective sleep alterations in mTBI patients compared with controls, particularly in the acute phase, with longer time in bed, reduced sleep efficiency, and increased nocturnal awakenings. Although these parameters tended to improve over time, residual increases in wake after sleep onset and awakening events persisted at 3 months, suggesting that mTBI may induce long-lasting fragmentation of sleep continuity, even in the absence of marked differences in total sleep time (Grima et al., 2016; Raikes and Schaefer, 2016). Actigraphy cannot determine sleep staging; therefore, our findings indicate fragmentation of sleep continuity rather than alterations in sleep architecture per se.

A novel and relevant finding was the presence of consistent, though modest, correlations between subjective and objective measures of sleep in mTBI patients. At 1 week, higher Epworth scores correlated with longer sleep latency and greater nocturnal fragmentation, while higher PSQI total scores were associated with reduced sleep efficiency and more awakenings. At 3 months, Epworth scores remained linked to actigraphic indices of fragmentation, and the PSQI sleep duration component correlated with reduced time in bed, shorter total sleep time, and more awakenings. These associations (ρ = 0.37–0.47) indicate that subjective complaints in mTBI are aligned with measurable disruptions in sleep continuity and efficiency. This pattern contrasts with reports in insomnia and non-TBI populations, where subjective and objective indices often diverge (Carskadon et al., 1976; Harvey and Tang, 2012; Buysse et al., 2008). Possible mechanisms include altered sleep perception, comorbid pain or mood disturbances, or dissociation between macrostructural and microstructural sleep features not captured by actigraphy alone (Chaput et al., 2009). Clinically, these findings support a multimodal assessment approach, as reliance solely on questionnaires or objective measures may underestimate or mischaracterize the true burden of post-traumatic sleep disturbance.

We operationalized subjective sleep disturbance as ESS >10 and/or PSQI >5. These thresholds derive from the original development papers and subsequent psychometric validations, where ESS >10 indexes clinically relevant daytime sleepiness and PSQI >5 discriminates poor sleep quality with acceptable accuracy. The instruments tap complementary constructs (daytime sleep propensity vs. nocturnal quality), and evidence indicates that their combined use improves detection of distinct sleep disturbance phenotypes in community and clinical samples (Buysse et al., 1989; Chaput et al., 2009; Thorarinsdottir et al., 2019). We therefore consider these cut-offs appropriate for defining patient-reported sleep disturbance in mTBI, while acknowledging known limitations of ESS in specific populations and the need to interpret scores alongside objective measures (Gurkan et al., 2024).

Hormonal analyses revealed elevated acute orexin A and B concentrations in patients who later developed persistent disturbances, along with lower orexin B in patients at 3 months. Orexinergic activation may promote wakefulness and contribute to sleep fragmentation. From a mechanistic perspective (Willie et al., 2001; Mahmood et al., 2004), persistent sleep fragmentation after mild TBI may reflect microstructural or inflammatory disruption within the hypothalamic and brainstem circuits that regulate sleep–wake balance. The ventrolateral preoptic nucleus (VLPO) promotes sleep through GABA/galanin inhibition of monoaminergic arousal centers (Ma et al., 2023), whereas the lateral hypothalamic orexin neurons stabilize wakefulness by providing excitatory input to those same nuclei (locus coeruleus, dorsal raphe, tuberomammillary) (Bouaouda and Jha, 2023). Injury-related reduction of VLPO inhibitory tone, combined with sustained or dysregulated orexin signaling, could therefore shift the arousal–inhibition balance toward hyperactivation, manifesting as shortened sleep latency and increased nocturnal awakenings. Additionally, the suprachiasmatic nucleus (SCN)—the master circadian pacemaker—modulates both melatonin secretion and VLPO activity (Comai and Gobbi, 2024); diffuse axonal injury or metabolic dysfunction within this network may impair circadian entrainment, leading to altered melatonin profiles and circadian misalignment. Collectively, these mechanisms offer a neurobiological framework linking hypothalamic–brainstem circuit vulnerability to the persistent hyperarousal and sleep–wake instability observed after mild TBI.

Interestingly, in controls subjective sleep complaints were not reflected in actigraphy but were associated with a distinct hormonal profile (higher morning melatonin and orexin B, lower orexin A), suggesting that neuroendocrine alterations may underlie subjective poor sleep even in the absence of objective fragmentation.

From a predictive perspective, we found that shorter minimal sleep latency at one week was the only independent predictor of persistent sleep disturbance at three months. This supports the concept that early objective markers of hyperarousal or impaired sleep onset stability may forecast chronic insomnia-like trajectories after mTBI. Importantly, similar patterns have been reported in larger prognostic cohorts of mild TBI. In the UPFRONT study (van der Naalt et al., Lancet Neurology, 2017), early post-injury features assessed within two weeks—including cognitive and emotional symptoms—were shown to predict long-term outcomes, reinforcing the idea that early intermediate markers can capture patients at risk for chronic sequelae. Our results extend this notion specifically to the sleep domain, highlighting minimal sleep latency as a candidate early biomarker of vulnerability (Mahmood et al., 2004; Zeitzer et al., 2009; van der Naalt et al., 2017).

Strengths include the prospective design, integration of subjective, objective, and biochemical measures, and a well-defined control group. This study has several important limitations that must be acknowledged. Primarily, the small sample size (n = 51 mTBI patients) failed to reach our a priori calculated target of 132 participants. This low recruitment over a three-year period was largely due to the high logistical burden placed on patients (e.g., wearing and returning actigraphs twice, undergoing multimodal assessments) and the lingering impacts of the COVID-19 pandemic. Consequently, our study is statistically underpowered to detect subtle differences, particularly regarding some hormonal biomarkers (e.g., melatonin), increasing the risk of Type II errors. Furthermore, the low recruitment rate raises the possibility of selection bias, meaning our cohort might not fully represent the broader mTBI population. Another limitation is the high proportion of control subjects presenting with pathological questionnaire scores; while this may reflect the increasingly high prevalence of poor sleep quality in the general population—particularly among healthcare workers and caregivers—it is important to note that active use of hypnotic medications was strictly excluded for all participants. Additionally, we lacked pre-injury sleep assessments for the mTBI cohort and did not systematically control for all potential confounders, such as pre-injury employment status, psychiatric history, or acute medication use, which could influence sleep architecture. Finally, the well-known high variability in actigraphy data and the inherent subjective biases of self-report measures reinforce the need to interpret our findings with caution.

Future research should address the mechanistic links between orexin dysregulation, sleep fragmentation, and recovery trajectories after mTBI, ideally combining actigraphy with polysomnography and neuroimaging of hypothalamic structures. Moreover, given the discrepancy between patients’ complaints and actigraphic data, treatment decisions should incorporate both domains to avoid undertreatment or overtreatment.

## 5 Conclusions

1.More than half of patients with mTBI experience persistent subjective sleep disturbances 3 months after injury, with objective evidence of ongoing nocturnal fragmentation.
2.Agreement between subjective questionnaires and actigraphy is limited, highlighting the need for combined assessment methods in both research and clinical practice.
3.Elevated acute orexin levels are associated with later persistence of sleep disturbance, but their predictive value is secondary to that of minimal sleep latency.
4.Short minimal sleep latency at 1 week is an independent predictor of persistent sleep disturbance, and may serve as a simple, early clinical marker for identifying high-risk patients.
5.Future studies should explore targeted interventions for patients with early hyperarousal, such as Cognitive Behavioral Therapy for Insomnia (CBT-I) or the use of targeted pharmacological agents like dual orexin receptor antagonists. Furthermore, investigating the mechanistic links between orexin dysregulation, chronic post-traumatic sleep pathology, and structural or functional neuroimaging (MRI) findings remains a critical next step to fully understand the neurobiology of mTBI recovery.

## Transparency, rigor, and reproducibility statement

The study design, inclusion criteria, and analysis plan were defined a priori and approved by the local Institutional Review Board (CEIC No. 21/391). Patient enrollment and follow-up were conducted prospectively, with predefined time points (1 week and 3 months). Clinical scales and actigraphy were applied using standardized, validated protocols. Biological samples were processed following established laboratory procedures, blinded to clinical outcomes. Statistical analyses were prespecified, and both parametric and nonparametric tests were applied consistently according to data distribution. All significant findings are reported transparently, including effect sizes, correlation coefficients, and p-values. Data integrity was ensured by independent data checking, and all analyses were reproducible using the documented dataset. Data supporting the main findings are available from the corresponding author upon reasonable request.

## Author contributions

Igor Paredes conceived and designed the study, coordinated patient recruitment, performed the analyses, drafted the manuscript, and is the guarantor of the work. Juan Delgado-Fernandez, Luis Miguel Moreno, Guillermo García-Posadas, Mónica Maldonado-Luna, Andreea Emanuela Baciu, and Leandro Tosi contributed to patient enrollment and clinical data collection. Victoria Cunha Alves and Cristina Sánchez Carabias collected and processed biological samples. Ana María Castaño-Leon and Alfonso Lagares provided statistical support, critical manuscript revision, and supervision of data interpretation. All authors reviewed and approved the final version of the manuscript and agree to be accountable for all aspects of the work.

## Declaration of AI use

During the preparation of this work, the authors used ChatGPT (OpenAI) to assist in improving the clarity, coherence, and linguistic accuracy of the manuscript. After using this tool, the authors critically reviewed and edited all generated content and take full responsibility for the scientific accuracy and integrity of the final version.

## Funding

This research received financial support from 10.13039/100008061Fundación Mutua Madrileña (Grant 2021).

## Conflict of interest

The authors declare that they have no conflicts of interest related to this work.

## Appendix A Supplementary data

Supplementary Table 1 Morning hormonal biomarkers in patients with mild traumatic brain injury (mTBI) and matched controls. Values represent mean ± standard deviation for serum and salivary melatonin, and serum orexin A and orexin B concentrations measured at 1 week and 3 months post-injury. Between-group comparisons were performed using Student's *t*-test or Mann–Whitney *U* test depending on data distribution. Significant differences are indicated by *p* < 0.05. Supplementary Table 1
