# Supplementary material for: Persistent sleep disturbances after mild traumatic brain injury: A prospective multimodal assessment with actigraphy and hormonal biomarkers
Source: Brain Spine. 2026 Mar 23;6:106014. doi: 10.1016/j.bas.2026.106014 (PMC13049660; doi:10.1016/j.bas.2026.106014)
Supplement: Supplementary Table 1 — Morning hormonal biomarkers in patients with mild traumatic brain injury (mTBI) and matched controls. Values represent mean ± standard deviation for serum and salivary melatonin, and serum orexin A and orexin B concentrations measured at 1 week and 3 months post-injury. Between-group comparisons were performed using Student's t-test or Mann–Whitney U test depending on data distribution. Significant differences are indicated by p < 0.05. [file mmc1.docx]

| **Supplementary Table 1. Morning Hormonal Biomarkers: mTBI vs Controls** | | | |
| --- | --- | --- | --- |
| Biomarker | mTBI (mean ± SD) | Controls (mean ± SD) | p-value |
| \| **1 Week Post-Injury** | | | |
| Serum Melatonin (pg/mL) | 99.90 ± 47.97 | 103.38 ± 68.45 | 0.805 |
| Salivary Melatonin (pg/mL) | 26.65 ± 28.43 | 17.50 ± 25.72 | 0.410 |
| Serum Orexin A (pg/mL) | 0.21 ± 0.14 | 0.21 ± 0.15 | 0.984 |
| Serum Orexin B (pg/mL) | 235.34 ± 99.59 | 257.55 ± 139.86 | 0.430 |
| **3 Months Post-Injury** | | | |
| Serum Melatonin (pg/mL) | 96.39 ± 32.01 | 104.31 ± 69.26 | 0.544 |
| Salivary Melatonin (pg/mL) | 19.90 ± 25.91 | 18.46 ± 26.17 | 0.877 |
| Serum Orexin A (pg/mL) | 0.22 ± 0.23 | 0.21 ± 0.16 | 0.802 |
| Serum Orexin B (pg/mL) | 218.46 ± 93.16 | 272.65 ± 105.48 | 0.041 |
